# Assessment of the Efficacy of Nalbuphine as an Adjuvant to Intrathecal Bupivacaine in Endoscopic Urological Surgeries for the Prolongation of Postoperative Analgesia

**DOI:** 10.7759/cureus.64257

**Published:** 2024-07-10

**Authors:** Saiesh Raut Dessai, Sanjot Ninave, Neeta Verma, Amol Bele, Aishwarya Nayak

**Affiliations:** 1 Department of Anaesthesiology, Jawaharlal Nehru Medical College, Datta Meghe Institute of Higher Education and Research, Wardha, IND; 2 Department of Anaesthesiology, Jawaharlal Institute of Postgraduate Medical Education and Research, Wardha, IND

**Keywords:** endourology, urology, 0.5% bupivacaine, nalbuphine, spinal anaesthesia

## Abstract

Background

In anaesthesiology, intrathecal drugs play pivotal roles in spinal anaesthesia. Despite their ability to induce a high sensory block, bupivacaine alone may not be adequate for postoperative analgesia. It often requires a substantial dose of postoperative rescue analgesia to manage pain effectively. Thus, we studied the efficacy of nalbuphine 1.5 mg injected intrathecally as an adjuvant in endoscopic urological surgery.

Materials and methods

Sixty patients undergoing endoscopic urological surgery were equally divided into two study groups: group B (injection 0.5% hyperbaric bupivacaine 15 mg (3 ml) plus sterile NS 0.15 ml) and group N (injection 0.5% hyperbaric bupivacaine 15 mg (3 ml)+nalbuphine 1.5 mg (0.15 ml)). The first appearance of the sensory and motor blockages and duration required to attain complete sensory and motor threshold was noted. All vitals were recorded. After surgery, it was recorded when the patient first needed rescue analgesia (injection paracetamol 1 gm IV). Any adverse effects were recorded and addressed. The statistical analysis was conducted using IBM SPSS Statistics for Windows, V. 22.0 (IBM Corp., Armonk, NY), with p<0.05 indicating significance in both groups' operations.

Results

Intrathecal nalbuphine as an adjuvant caused an earlier onset of sensory and motor inhibition, delayed two-segment regression, and prolonged postoperative anaesthesia. The control group experienced sensory block at 3.33±0.61 minutes, while the nalbuphine group had a mean onset of 2.66±0.92 minutes (p=0.001). The patient who received nalbuphine had a mean regression time of 119.60±14.549 minutes, whereas the bupivacaine group had a mean regression time of 88.43±17.196 minutes. Group N had a considerably longer duration of postoperative analgesia, lasting 264.97 minutes, compared to group B's 198.50 minutes (p<0.001). Intrathecal nalbuphine did not influence vital indicators such as heart rate, respiration rate, and oxygen saturation.

Conclusion

To conclude, endoscopic urological surgery patients who received a subarachnoid block with 1.5 mg (0.15 ml) of nalbuphine hydrochloride with 0.5% hyperbaric bupivacaine 15 mg (3 ml) had longer postoperative pain relief than those who received 3 ml of intrathecal bupivacaine (15 mg). Urinary retention and pruritus were absent. Intrathecal nalbuphine with hyperbaric 0.5% bupivacaine is deemed safe with minimal side effects in endoscopic urology surgery.

## Introduction

In anaesthesiology, intrathecal drugs play pivotal roles in spinal anaesthesia, especially for procedures affecting the lower extremities, pelvis, and lower abdomen. Several types of local anaesthetics are used in different types of anaesthesia, such as epidural, spinal, regional, and local infiltration. Examples of local anaesthetics include bupivacaine and lidocaine, which work by obstructing the transmission of nociceptive signals by inhibiting voltage-gated sodium channels [1].

Bupivacaine is an amide-group local anaesthetic. It is helpful in clinical practice due to its unique qualities. Morphine and fentanyl are opioids that bind to opioid receptors in the spinal cord [2]. Adjuvants such as clonidine and dexmedetomidine enhance analgesia by several means, such as N-methyl-D-aspartate (NMDA) receptor inhibition and α2-adrenergic agonism [3,4]. Compared to other analgesia methods, intrathecal anaesthesia reduces pain with fewer systemic adverse effects. Intrathecal medication administration has several advantages, but it is not without its problems. Thorough dosing and monitoring are necessary to avoid adverse effects like hypotension, respiratory depression, and neurotoxicity [5]. Despite its ability to induce a high sensory block, bupivacaine alone may not be adequate for postoperative analgesia. It often requires a substantial dose of postoperative rescue analgesia to manage pain effectively, e.g., paracetamol.

Nalbuphine has excellent solubility in lipids and functions as an opioid analgesic. It acts as an agonist at the κ-opioid receptor, and it also functions as an antagonist. This unique mechanism allows it to offer potent analgesia for visceral nociception [6]. Nalbuphine is often used for both local and general anaesthesia procedures. Combining hyperbaric bupivacaine with this adjuvant enhances perioperative analgesia with minimal adverse effects [7]. Nalbuphine enhances the function of κ-opioid receptors while decreasing the function of μ-opioid receptors [8]. Hence, the study aimed to evaluate the effectiveness of administering 1.5 mg of nalbuphine intrathecally as an adjuvant during endoscopic urological surgery.

## Materials and methods

Study design, setting, ethical consideration, and participants

The present prospective randomised controlled trial was carried out among 60 patients who were undergoing endoscopic urological surgeries and were scheduled for subarachnoid block after an appropriate preanaesthetic check-up. The study was carried out in the Department of Anaesthesiology, Jawaharlal Nehru Medical College, Wardha, India. The study was commenced after the Institutional Ethics Committee of Datta Meghe Institute of Higher Education and Research approved it (approval number: DMIMS(DU)/IEC/2022/93). Before the procedure, all patients willing to participate were informed about the study and procedure involved, and appropriate consent was obtained in the prescribed format. A total of 60 patients, fulfilling all the inclusion criteria, were enrolled in the present study and were randomly allocated into two study groups: group B (n=30) (injection hyperbaric bupivacaine 15 mg (3 ml)+sterile NS 0.15 ml) and group N (n=30) (injection hyperbaric bupivacaine 15 mg (3 ml)+injection nalbuphine 1.5 mg (0.15 ml)). The statistical analysis was carried out with p<0.05 considered as significant in the procedures carried out by the two groups.

Study criteria (inclusion and exclusion)

Inclusion criteria comprised males and females in the age group of 20-70 years, patients undergoing urological surgeries, American Society of Anesthesiologists (ASA) physical status classification systems I and II, Mallampati classification (MPC) scores I and II, patients willing to participate in the study, and patient fulfilling criteria for subarachnoid block. Exclusion criteria comprised lack of valid informed written consent, ASA grades III and IV, infection at the subarachnoid block injection site, patients with neurological and musculoskeletal disease, patients with bleeding disorders, patients on anticoagulants, history of allergy to local anaesthetic and injection of nalbuphine, increased intracranial pressure, and spinal deformity.

Procedure

A detailed history, general physical, and systemic examination were performed on patients posted for endoscopic urological surgeries. Blood tests, chest X-rays, and electrocardiograms (ECGs) performed by treating clinicians were reviewed. The physical status was evaluated to determine the ASA grade, and the airway was evaluated to determine the MPC grade. The spinal anaesthesia procedure and the drugs to be used were thoroughly explained to the patients. Patients were kept nil per oral for six hours prior to the surgery. Before surgery, the patient received a detailed explanation of the anaesthetic method and the visual analogue scale (VAS) for pain assessment. They were then asked to provide their informed permission. The predesigned performa collected data such as the patient's age, weight (in kilograms), ASA grade, and surgical indication.

A multipara monitor was fastened to the patient upon arrival in the operating room in order to capture critical data such as electrocardiography, non-invasive blood pressure, oxygen saturation, respiratory rate, and heart rate. The patient had an 18 G intravenous cannula placed, and 10 ml/kg Ringer lactate was preloaded. A continuous visual display of a five-lead ECG was confirmed, and the following parameters such as heart rate, respiratory rate, non-invasive blood pressure, and oxygen saturation were assessed. Ten minutes before anaesthesia, all individuals were intravenously premedicated with 75 mcg/kg of ondansetron. A subarachnoid block was performed in a left lateral posture using a 25-gauge Quincke's spinal needle at the L3-4/L4-5 intervertebral area, following all aseptic precautions. After ensuring the free flow of the cerebrospinal fluid (CSF), the study drug was injected. The patient was turned to a supine position, and a pillow was provided below the shoulder. To check for sensory blockade, the pinprick technique was used, which included a blunt-tipped needle. The modified Bromage scale was used to evaluate the quality of motor blockade, and surgery was allowed to proceed [6].

The initial onset of sensory and motor blockade, along with the time needed to achieve complete sensory and motor threshold, was recorded. The patient's level of sedation was assessed using the modified Ramsay sedation scale. At five, 10, and 15 minutes into the operation and then every 15 minutes after that, the procedure was finished. The highest sedation score was recorded. Beat-to-beat monitoring was performed for heart rate; electrocardiography, oxygen saturation, and blood pressure (both systolic and diastolic) were recorded every two minutes until 10 minutes, every 15 minutes until 30 minutes, and, lastly, every 30 minutes until the operation is over. Adverse effects, if any, were noted and managed accordingly. If heart rates fell to 60 beats per minute (bradycardia) or 30% below their baseline rates, intravenous glycopyrrolate 0.2 mg was administered. Intravenous 3 mg mephentermine was given for a decrease in blood pressure below 20% of baseline. Injection ondansetron 4 mg was administered if the patient complained of nausea and vomiting. For shivering, an intravenous injection tramadol 50 mg was administered.

## Results

A total of 60 participants were recruited and were analysed. Both groups were comparable with respect to demographic data with no statistically significant difference (Table 1).

**Table 1:** Comparison of demographic data between two groups M: males; F: females

Parameters	Group N	Group B	P-value
Age (years)	50.16±15.50	57.10±7.40	0.383
Sex (M:F)	21:9	19:11	0.73
Weight (kgs)	60.33±8.96	53±12.69	0.52
Height (cms)	162.2±4.34	162.7±5.03	0.56

The onset of sensory block was non-similar across the two groups, with a statistically significant difference (p=0.001) and faster onset of sensory block with nalbuphine as an adjuvant. The motor blockade onset was non-similar across the two groups, but participants who received nalbuphine attained a mean onset of motor block faster compared to bupivacaine alone. The mean duration of analgesia was higher in nalbuphine as compared to bupivacaine, with a statistically significant difference (Table 2).

**Table 2:** Characteristics of the blockade

Parameters (minutes)	Group N	Group B	P-value
Onset of sensory block	2.66±0.92	3.33±0.61	0.001
Onset of motor block	3.56±0.93	5.03±0.72	0.001
Time for two-segment regression	152.47±6.47	128.13±6.01	0.001
Total duration of analgesia	264.97±13.20	198.50±14.54	0.001

Hemodynamic parameters were comparable across both groups. Five participants in group N and two participants in group B had episodes of hypotension with two incidences of bradycardia in group N and one in group B. Hypotension was treated by administering bolus 100-200 ml colloid solution, preferably Ringer lactate. No participants with hypotension required vasopressor support. Four patients in the study group and two participants in the control group had shivering, which was managed by injection tramadol 25 mg IV. No participants complained of pruritus, sedation, and breathing difficulty (Table 3).

**Table 3:** Side effects observed in both groups

Postoperative complications	Group N	Group B	P-value
Nausea	3	1	0.612
Hypotension	5	2	0.406
Bradycardia	2	1	0.879
Pruritus	0	0	1.000
Shivering	4	2	0.564
Respiratory depression	0	0	1.000

## Discussion

Spinal anaesthesia has been the subject of many studies to refine the technique and enhance patient outcomes using different medications. In order to prolong the duration and ensure the early onset of the anaesthetic effects, adjuvants are often delivered intrathecally with hyperbaric bupivacaine 0.5%. They exert their analgesic effects by acting on receptors present on the spinal cord. Adjuvant intrathecal opioids may cause sensory and motor blockage to begin early and persistent postoperative analgesia. Because of their sympathetic and motor-sparing actions, they also enable patients to walk early. The results of this prospective randomised controlled trial showed that 1.5 mg of nalbuphine as an adjuvant to hyperbaric bupivacaine caused sensory and motor blockade to begin earlier and the duration of analgesia to last longer. The bupivacaine group experienced sensory block at 3.33±0.61 minutes, compared to the nalbuphine group which had a mean duration of 2.66±0.92 minutes. One hundred participants undergoing spinal anaesthesia for lower limb orthopaedic operations were compared by Mukherjee et al. who concluded that the motor blockade onset of different doses of nalbuphine were not statistically significant when combined with 0.5% hyperbaric bupivacaine [9]. Statistical analysis of a comparable trial by Roy et al. compared nalbuphine as an adjuvant to bupivacaine and bupivacaine alone for abdominal hysterectomy, and the results showed that nalbuphine significantly induced faster motor block [10].

The average onset time of motor block in bupivacaine group B was 5.03±0.72 minutes, and group N had an average of 3.56±0.93 minutes. The nalbuphine group had faster sensory and motor block onset times, according to statistical analysis. Roy et al. [10] compared nalbuphine and bupivacaine for abdominal hysterectomy statistically. Nalbuphine appeared to cause motor block faster. Mehta et al. [11] discovered that nalbuphine as an adjuvant to bupivacaine caused sensory and motor blockades faster. Ahmed et al. [12] concluded that the onset of motor blockade between 0.8 mg, 1.6 mg, and 2.4 mg nalbuphine were not statistically significant. Tiwari et al. [13] concluded that intrathecal nalbuphine did not have a significant effect on motor and sensory block. Borah et al. and Mukherjee et al. found similar results [9,14]. It was found that both groups achieved the same sensory blockage at various post-procedure periods without statistically significant differences. Compared to the control group, more patients given nalbuphine could reach a higher sensory level (T4). Deori et al. [15] also discovered similar results, with no statistically significant difference in the maximum degree of sensory block achieved.

Statistically, there was no significant difference (p>0.05) in hemodynamic variable rates between the two groups during surgery. The hemodynamic findings in researches conducted by Satapathy et al. [16] and Sharma et al. [17] indicated no notable hemodynamic difference between nalbuphine and bupivacaine in lower limb procedures. Sapate et al. [18] found a significant difference in the hemodynamic profile between the nalbuphine and control groups. The group that received nalbuphine had elevated average heart rates and increased systolic and diastolic blood pressures. Sharma et al. [17] found that the blood pressure and heart rate were comparable when comparing bupivacaine to nalbuphine and fentanyl. The two-segment regression of the sensory block for the nalbuphine group and bupivacaine group were 152.47 and 128.13 minutes, respectively. The nalbuphine group had significantly slower two-segment regression than the bupivacaine group. Deori et al. [15] observed that the group that received nalbuphine had prolonged regression time. The findings of this investigation are corroborated by Borah et al. [14]. The lipophilic nature of nalbuphine likely accounts for its lightning-fast analgesic effects. A recent study showed that sensory and motor blocks developed at distinct rates, with sensory blocks regressing more slowly. Shah et al. [19] found a statistically significant difference in the total duration of analgesia between the control and nalbuphine groups (p<0.001). Amin et al. [20] and Borah et al. [14] reached comparable findings.

Adverse effects such as bradycardia, hypotension, nausea, pruritus, shivering, respiratory distress, and other complications were not statistically significant between the two groups. Furthermore, no one who participated in the trial had a headache. This could be because the research individuals were appropriately informed before inducing the subarachnoid block and a 25-gauge spinal needle was utilised. Since our standard procedure involved preloading all research participants with 10 ml/kg of Ringer lactate before the spinal block to avoid hypotension, the current investigation did not find a statistically significant case of hypotension. Fewer patients in the nalbuphine group reported postoperative nausea and vomiting (PONV) (p=0.008) in a study by Liu et al. [21]. In their study, Borah et al. looked at nalbuphine dosages ranging from 0.4 to 1.6 mg. They discovered that 0.8 and 0.4 mg both provide excellent analgesia, while 1.6 mg causes side effects such as nausea and vomiting [14]. Because the nalbuphine opioid receptors were partly antagonised and, thus, attenuated, the current investigation found no evidence of pruritus in the study group.

It was observed in the present study that postoperative pain scores were much lower with intrathecal nalbuphine as an adjuvant when compared with intrathecal bupivacaine alone, according to the current investigation. The VAS score of 4 was considered as the termination of analgesia. When the patients had a VAS score of 4, rescue analgesic (1 g IV paracetamol) was given. Compared to the bupivacaine group, the nalbuphine group had superior postoperative analgesia. The p-value is less than 0.001. In the present study, the nalbuphine group had a considerably longer duration of postoperative analgesia, lasting 264.97 minutes, compared to the bupivacaine group which was 198.50 minutes (p<0.001). The findings of our study are consistent with those of Borah et al. [14], Amin et al. [20], and Tiwari et al. [13], all of which found that nalbuphine prolonged the analgesia. Patients may be able to tolerate early ambulation and be discharged from the hospital sooner if their postoperative pain is controlled. Nociceptive sensations are modulated and processed by the spinal cord. Intrathecal opioids are helpful for prolonging postoperative pain management. This study adds to the current knowledge on the use of nalbuphine intrathecally, but the results should be reviewed taking into consideration the various limitations. Firstly, all the study subjects were either ASA I or II. Secondly, the impact on sensory level after position change should have been considered. Thirdly, the study was carried out on a limited number of study subjects (n=60).

## Conclusions

The study discovered that patients who underwent endoscopic urological surgery and were given a subarachnoid block with 1.5 mg of nalbuphine experienced a faster onset of sensory and motor inhibition, delayed two-segment regression, and prolonged postoperative anaesthesia than those who received 3 ml of intrathecal bupivacaine (15 mg) alone.
